# Correction to “Sex‐Related Differences in Physiologically‐Based Biopharmaceutics Modeling”

**DOI:** 10.1002/psp4.70324

**Published:** 2026-08-12

M. Chavarría‐Rojas , M. Murshed , M. Lorier , N. Fotaki , and M. Ibarra . “Sex‐Related Differences in Physiologically‐Based Biopharmaceutics Modeling,” CPT: Pharmacometrics & Systems Pharmacology 15 (2026): e70310, 10.1002/psp4.70310.42528013 PMC13580755

In the article cited above, the authors inadvertently omitted acknowledgment of the academic software licenses provided by Certara UK Limited (Simcyp) and Simulations Plus, Inc. (GastroPlus) without which this work would not have been possible. The authors sincerely regret this omission.

The correct Acknowledgments section is shown below.

